# Copper-based nanozymes synergistically enhance Cuproptosis for psoriasis treatment

**DOI:** 10.1016/j.mtbio.2026.102855

**Published:** 2026-01-29

**Authors:** Junyu Zhou, Nianzhou Yu, Xiaoxin Yang, Shanghong Li, Mi Huang, Tianyi Pang, Dong Zhong, Yu Wen, Hong Liu

**Affiliations:** a Central South University (Department of Dermatology, Xiangya Hospital, Furong Laboratory), Changsha, Hunan, 410008, China; b National Engineering Research Center of Personalized Diagnostic and Therapeutic Technology, China; c National Clinical Research Center for Geriatric Disorder, Xiangya Hospital, Changsha, Hunan, 410008, China; d Hunan Key Laboratory of Skin Cancer and Psoriasis, Xiangya Hospital, Changsha, Hunan, 410008, China; e Hunan Engineering Research Center of Skin Health and Disease, Xiangya Hospital, Changsha, Hunan, 410008, China; f Department of Nuclear Medicine, Xiangya Hospital, Central South University, Changsha, Hunan, 410008, China

**Keywords:** Psoriasis, Cuproptosis, Nanozyme, Treatment

## Abstract

Psoriasis is a chronic inflammatory skin disease characterized by abnormal keratinocyte proliferation and sustained skin inflammation. Cuproptosis, a novel regulated cell death pathway, inhibits proliferation by promoting cellular demise, offering a promising therapeutic strategy for psoriasis. Herein, we first identified that cuproptosis induction is a potential therapeutic avenue for psoriasis treatment. Then, a copper-based nanozyme (Cu-NZ) was developed to enhance cuproptosis through cascade catalytic therapy, leveraging multi-enzymatic effects for psoriasis treatment. The Cu-NZs exhibited distinct multi-enzymatic activities, including catalase (CAT)-, superoxide dismutase (SOD)-, oxidase (OXD)-, and peroxidase (POD)-like activities, which sustained the generation of cytotoxic Reactive Oxygen Species (ROS), relieved hypoxia via O_2_ release, and ultimately triggered augmented cuproptosis. In vitro results demonstrated that Cu-NZs suppressed HaCaT cells proliferation and inflammatory factor expression while inducing mitochondrial dysfunction through ROS elevation. Mechanistically, Cu-NZs modulated the expression of cuproptosis-related genes and proteins (DLAT, FDX1, LIAS). In vivo studies confirmed that topical Cu-NZs gel significantly alleviated imiquimod (IMQ)-induced psoriatic phenotypes in mice without inducing systemic organ toxicity. Collectively, Cu-NZs mitigated psoriasis manifestations by triggering cuproptosis in keratinocytes, thereby inhibiting their pathological activation and proliferation. These findings provided a theoretical foundation for the clinical translation of Cu-NZs-based therapies.

## Graphical abstract

**Figure:**
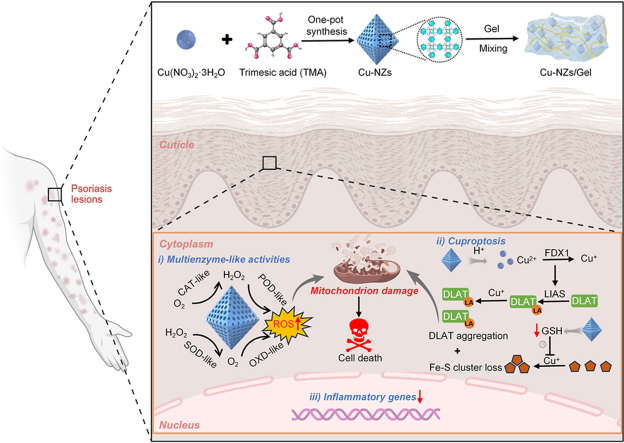

## 1 Introduction

Psoriasis is a common, chronic, systemic, immune-mediated inflammatory skin disease. It is frequently associated with diverse extracutaneous manifestations, including involvement of the joints, cardiovascular system, and metabolic system. Its global prevalence is estimated at 1–5 %, affecting over 60 million individuals worldwide [1]. The pathological hallmarks of psoriasis are characterized by aberrant keratinocyte hyperproliferation, impaired epidermal differentiation, and immune cell infiltration. This is accompanied by the excessive release of pro-inflammatory cytokines such as TNF-α, IL-17, and IL-23 [2]. Although the development of biologic agents (e.g., IL-17 monoclonal antibodies) and small-molecule targeted therapeutics has significantly improved clinical outcomes, considerable challenges remain [3,4]. These include disease recurrence, heterogeneous treatment responses among individuals, and unresolved long-term safety concerns.

In recent years, metabolic reprogramming and dysregulated cell death modalities have been demonstrated to play pivotal roles in the pathogenesis of psoriasis [[5], [6], [7]]. In the skin microenvironment, an imbalance between oxidative stress and antioxidant defense is an important underlying mechanism for the occurrence and progression of psoriasis [8]. Reactive Oxygen Species (ROS) accumulation – resulting from mitochondrial dysfunction, nicotinamide adenine dinucleotide phosphate (NADPH) oxidase (NOX) activation, and diminished antioxidant enzymes (such as superoxide dismutase and glutathione (GSH) peroxidase) – serves as a potent secondary messenger [9,10]. This pathway constitutes a particularly critical mechanistic component in psoriasis.

In 2022, the discovery of cuproptosis, a novel copper-dependent form of regulated cell death, has provided a new perspective for investigating the mechanisms underlying metabolic diseases [11]. As it is significantly distinct from other known cell death pathways, such as necroptosis, pyroptosis, ferroptosis and apoptosis [12]. This process is triggered by excessive copper ions directly binding to lipoylated components of the tricarboxylic acid (TCA) cycle, particularly the key proteins FDX1 (the most upstream regulatory protein responsible for Fe-S cluster loss) and DLAT [[13], [14], [15]]. This binding leads to their aggregation, inducing proteotoxic stress and ultimately cell death. Its regulatory core centers on the mitochondrial respiratory chain and energy metabolism pathways [16]. Notably, several studies have reported significantly elevated copper ion concentrations within psoriatic lesions. Concurrently, dysregulated expression of copper homeostasis regulators has been observed [17,18]. These findings suggest a potential association between disrupted copper metabolism and disease progression. Meanwhile, bioinformatics studies have reported the diagnostic and subtyping significance of cuproptosis-related genes in psoriasis [19]. However, the fundamental role of cuproptosis in psoriasis pathogenesis remains unexplored, with its underlying mechanisms poorly understood. This knowledge gap presents a promising research avenue for developing cuproptosis-targeted therapeutic materials.

Cu-based nanozymes constitute a large family of oxidoreductase-mimicking catalysts that exhibit oxidase (OXD)-, peroxidase (POD)-, catalase (CAT)-, and superoxide dismutase (SOD)-like activities [20,21]. Notably, those with antioxidant capacity can effectively scavenge reactive oxygen species (ROS) and protect cells from oxidative damage, offering a promising therapeutic strategy for a broad range of diseases [22,23]. Given the physiological pH gradient of human skin—neutral at the surface and increasingly acidic in deeper layers, we hypothesize that transdermal delivery of Cu-based nanozymes to the neutral epidermis will unleash multi-enzyme catalytic cascades that suppress keratinocyte proliferation [24]. Selective induction of cuproptosis within the stratum corneum could interrupt the pathogenic feedback loop and provide a novel therapeutic avenue for psoriasis.

**Fig. 1:**
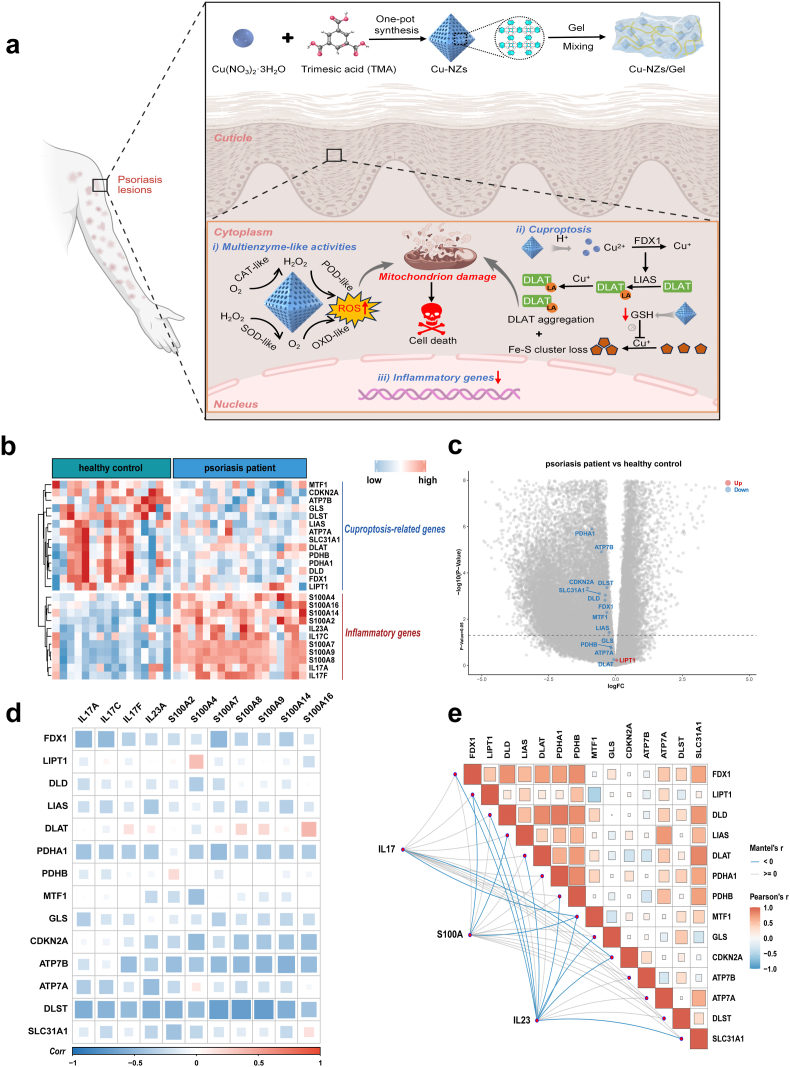
**Schematic of the preparation and therapeutic mechanism of Cu-NZs and bioinformatics analysis of cuproptosis-related genes in psoriasis.** (a) Schematic illustration of Cu-NZs synthesis and its mechanism of action in psoriatic keratinocytes. (b) Expression heatmap of 14 cuproptosis-related genes and inflammatory genes in healthy controls versus psoriasis patients. (c) Volcano plot showing differential expression of 14 cuproptosis-related genes in psoriasis patients compared to healthy controls. (d) Spearman correlation heatmap depicting associations between 14 cuproptosis-related genes and inflammatory genes. (e) Mantel test analysis of correlations between three psoriasis-associated inflammatory gene families (IL-17, S100A, IL-23) and 14 cuproptosis-related genes.

In this study, we first established cuproptosis induction as a novel therapeutic strategy for psoriasis, and then designed copper-based nanozymes (Cu-NZs) that were designed to trigger cuproptosis in keratinocytes for psoriatic therapy (Fig. 1a). These NZs exhibited intrinsic, cascade-like enzymatic activities – including CAT-, SOD-, OXD-, and POD-like properties – under mildly acidic conditions. Consequently, they continuously generated cytotoxic ROS, released O_2_ to relieve hypoxia, massively depleted endogenous antioxidant and copper-chelating pools (e.g., glutathione (GSH) and NADPH), and ultimately amplified cuproptosis. Upon traversing the skin and entering stratum corneum cells, the NZs generated abundant ROS that disrupted mitochondrial function, triggered cuproptosis hallmarked by loss of Fe–S cluster proteins plus aggregation of lipoylated proteins, and suppressed the expression of key inflammatory cytokines. In vivo results, the Cu-NZs gel achieved striking therapeutic efficacy by alleviating imiquimod (IMQ)-induced psoriatic phenotypes in mice without inducing systemic organ toxicity, demonstrating the therapeutic superiority of this Cu-based multi-enzymatic strategy.

## 2 Materials and methods

### 2.1 Bioinformatics analysis of cuproptosis-related genes in psoriasis

The publicly available dataset GSE74697 from the GEO database was conducted for data processing. It contained expression data for 18 patients with psoriasis and 16 healthy controls [25]. 14 copper-dependent cell death (cuproptosis)-related genes (*FDX1, LIPT1, DLD, LIAS, DLAT, PDHA1, PDHB, MTF1, GLS, CDKN2A, ATP7B, ATP7A, DLST* and *SLC31A1*) were identified based on previously reported literature [26,27]. Within the R (version 4.4.2), the normalization of the expression matrix from this dataset was performed using DESeq2 and conducted differential expression analysis between psoriatic patients and healthy controls. The correlations between inflammation-associated genes relevant to psoriasis pathogenesis and these cuproptosis-related genes were examined using both Spearman's rank correlation and Mantel tests. Visualization of the results was implemented employing the ComplexHeatmap, ggplot2, and corrplot packages.

### 2.2 Materials

Copper nitrate trihydrate (Cu(NO_3_)_2_•3H_2_O), benzene-1,3,5-tricarboxylate (TMA) PVP-K30, methanol, ethanol, N,N-Dimethylformamide (DMF), hydrogen peroxide (H_2_O_2_, 30 %), 3, 3′, 5, 5’ -Tetramethylbenzidine dihydrochloride hydrate (TMB), were obtained from Shanghai Titan Scientific Co., Ltd. The superoxide dismutase assay kit, and catalase (CAT) assay kit were obtained from Beyotime Institute of Biotechnology.

### 2.3 Structural characterizations

A transmission electron microscope (TEM, JEM-2100) was used to image samples at an acceleration voltage of 200 kV. The morphology of samples was also studied by using a scanning electron microscope (SEM, Zeiss Sigma 300). The Powder X-ray diffraction (PXRD) patterns of particles were collected on a Bruker D8 Q diffractometer with a scanning rate of 10°/min in the range of 50–90° with Ni-filtered Cu/K-α radiation (1.5418 Å). The hydrodynamic size and zeta protentional of samples were measured on a Malvern Zetasizer Nano ZSP. TGA-DC was performed using a Netzch 209F3 thermal analyzer system at a rate of 10 °C min^−1^ under N_2_ atmosphere. Fourier translation infrared (FT-IR) spectra were obtained on a Perkin Elmer Spectrum 100 spectrometer (Perkin Elmer) using the KBr pellet method. X-ray photoelectron spectroscopy (XPS) was collected using a multifunctional photoelectron spectrometer Axis Ultra DLD (Kratos, UK). The CAT-mimicking activity of samples were evaluated by detecting the O_2_ generation with a dissolved oxygen meter. The UV–visible spectra were obtained on a Shimadzu UV-3600 spectrophotometer. Electron spin resonance (ESR) spectra were acquired using a Bruker EMXplus ESR spectrometer.

### 2.4 Preparation of Cu-NZs (MOF-199)

BTC (0.1 M, 3 mL), Cu(NO_3_)_2_•3H_2_O (0.2 M, 0.5 mL) and 100 mg PVP-K30 were added to a 10 mL mixture of methanol and DI water (1: 1 v/v), followed by stirring at room temperature for 3 h (300 rpm). Subsequently, the reaction solution was centrifuged (11000 rpm, 15 min) and washed three times with DMF and ethanol, respectively. The resulting precipitate was sonicated in ethanol for further use.

### 2.5 Catalase (CAT)-mimicking activity of Cu-NZs

Typically, different concentrations of Cu-NZs (0, 25, 50, and 100 μg/mL) was added into H_2_O with different concentrations of H_2_O_2_ (50 mM), the dissolved oxygen concentration (mg/L) was immediately recorded for 300 s using a dissolved oxygen meter.

### 2.6 Superoxide dismutase (SOD)-mimicking activity of Cu-NZs

In a typical assay, the solution containing nitrotetrazolium blue chloride (NBT) (0.2 mM), xanthine (X) (0.3 mM) and xanthine oxidase (XO) (0.1 U/mL) was mixed with different concentrations of Cu-NZs (0, 25, 50, and 100 μg/mL) in Tris-HCl buffer (0.1 M, pH 7.2), then the absorbance at 550 nm of the mixed solution samples were continuously monitored in 10 min. The SOD-like activity of Cu-NZs was also tested through ESR measurements.

### 2.7 Oxidase (OXD)-mimicking activity of Cu-NZs

In a typical process, TMBTMB (50, 100, 200, 400 and 800 μM), and Cu-NZs (0, 5, 10, 20, and 40 μg/mL) were mixed in Tris-HCl buffer (0.1 M, pH 7.2). The absorbance was recorded after a certain reaction time using a UV–vis spectrophotometer and microplate reader (absorbance at 650 nm). Kinetic measurements of the interactions between Cu-NZs and TMB as a substrate were achieved at various concentrations of TMB.

### 2.8 Peroxidase (POD)-mimicking activity of Cu-NZs

In a typical process, TMB (50, 100, 200, 400 and 800 μM), H_2_O_2_ (0, 5, 25, 50, and 100 mM) and Cu-NZs (0, 5, 10, 20, and 40 μg/mL) were mixed in Tris-HCl buffer (0.1 M, pH 7.2). The absorbance was recorded after a certain reaction time using a UV–vis spectrophotometer and microplate reader (absorbance at 650 nm). Kinetic measurements of the interactions between Cu-NZs and TMB as a substrate were achieved at various concentrations of TMB and fixed concentration of H_2_O_2_. The POD-like activity of Cu-NZs was also tested through ESR measurements.

### 2.9 Density functional theory (DFT) calculations

All the DFT calculations were conducted based on the Vienna Ab-inito Simulation Package (VASP) [28,29]. The exchange-correlation effects were described by the Perdew-Burke-Ernzerhof (PBE) functional within the generalized gradient approximation (GGA) method [30,31]. The core-valence interactions were accounted by the projected augmented wave (PAW) method [32]. The energy cutoff for plane wave expansions was set to 400 eV, and the 1 × 1 × 1 Gamma centered k-points were selected to sample the Brillouin zone integration. The structural optimization was completed for energy and force convergence set at 1.0 × 10-4 eV and 0.08 eV Å-1, respectively. DFT-D3 method was used to describe van der Waals (vdW) interactions [33].

The Gibbs free energy changes (ΔG) of the reaction werecalculated using the following formula:

$$\Delta G=\Delta E+\Delta \text{ZPE}-T\Delta S+\Delta G_{U}+\Delta G_{\text{pH}}$$

where Δ*E* is the electronic energy difference directly obtained from DFT calculations, ΔZPE is the zero-point energy difference, *T* is the room temperature (298.15 K) and ΔS is the entropy change. ΔG_U_ = -e*U*, where *U* is the applied electrode potential. ΔG_pH_ = *k*_B_T × ln 10 × pH, where *k*_B_ is the Boltzmann constant, and pH value is set to 0.

The transition state were subsequently calculated by climbing image nudged elastic band (CI-NEB) methods.

### 2.10 Cell culture and treatment

The immortalized human keratinocyte line HaCaT (CellCook cat: CC4013) was maintained in Dulbecco's Modified Eagle Medium (DMEM; Gibco) enriched with 10 % fetal bovine serum (FBS; CellMax) and 1 % penicillin/streptomycin solution. Cell cultures were incubated at 37 °C under a humidified 5 % CO_2_ atmosphere. To establish an in vitro psoriatic inflammatory model, HaCaT cells were challenged with an M5 cytokine cocktail (10 ng/mL; TNF-α, IL-17A, IL-22, IL-1α, and Oncostatin-M; Sino Biological) for 24 h [34].

### 2.11 Cellular uptake

HaCaT cells (1 × 10^5^/well) were treated with 5 μg/mL ^ICG−^Cu-NZs for 0–48h. After each interval, cells underwent ice-cold PBS washes, trypsinization (Beyotimes; 0.25 %, 10 min), centrifugation (500×*g*, 5 min), and resuspension in FACS buffer (PBS/5 % FBS). For fluorescence quantification of cellular internalization, flow cytometry was performed using a BD LSRFortessa analyzer (Alexa Fluor 700 channel: 692ex/719em). Data from 10,000 single-cell events were analyzed using FlowJo software (v10.4), and the internalization level was reported as the median fluorescence intensity (MFI). For visual confirmation, confocal microscopy was conducted on fixed and DAPI-stained (10 min) samples using a Zeiss LSM980 microscope (Alexa Fluor 700: 692ex/719em).

### 2.12 CCK-8 assay

Cellular viability was assessed using the CCK-8 assay. HaCaT cells were plated in 96-well plates at 8000 cells/well. Following 24-h treatments with varying concentrations of Cu-NZs, cultures were incubated with 100 μL of fresh medium containing 10 μL CCK-8 reagent (Selleckchem) for 1 h at 37 °C/5 % CO_2_. Absorbance was subsequently recorded at 450 nm using a Bio-Rad Model 680 microplate reader.

### 2.13 Flow cytometric analysis of Cell proliferation

HaCaT cells were seeded in 6-well plates at 8 × 10^5^ cells/well for overnight adherence. Following 24-h Cu-NZs treatment, cells were harvested by trypsinization, washed with ice-cold phosphate-buffered saline (PBS), and fixed in 4 % paraformaldehyde (15 min,RT). Fixed cells were permeabilized with Foxp3/Transcription Factor Staining Buffer Set (eBioscience) according to manufacturer specifications (30 min, RT). Cells were incubated with BV650-conjugated anti-Ki-67 antibody (Biolegend; #151215; 1:200 dilution) for 30 min at RT protected from light. After washing, samples were resuspended in PBS. Flow cytometry was performed using a BD LSRFortessa™. Data were analyzed in FlowJo v10.4 quantified Ki-67 expression as MFI of positive populations.

### 2.14 Intracellular catalytic O_2_ production activity

To detect intracellular oxygen levels, the oxygen-sensitive fluorescent probe [Ru(dpp)_3_]Cl_2_ (Shanghai Maokang Biotechnology Co., Ltd.) was employed. The experimental procedure was consistent with the aforementioned method [35]. Briefly, after fixation, cells were treated with 10 μM [Ru(dpp)_3_]Cl_2_ and incubated in the dark for 30 min. Following DAPI staining, the samples were finally observed using Zeiss LSM980.

### 2.15 Intracellular ROS determination

Following overnight adherence in 6-well plates (8 × 10^5^ cells/well), HaCaT cells were exposed to Cu-NZs for 24 h. After medium aspiration, cells underwent trypsinization, centrifugation (500×*g*, 5 min), and two PBS washes. Pellets were respectively resuspended in DCFH-DA (Solarbio Life Sciences), DHE (Beyotime) or HPF (Shanghai Maokang Biotechnology Co., Ltd.) and incubated at 37 °C for 30 min in light-protected conditions. For confocal microscopy, Cells were then washed and immediately imaged using Zeiss LSM980. For flow cytometric analysis, intracellular ROS levels were quantified by measuring MFI in FlowJo v10.4 by using BD LSRFortessa™.

### 2.16 JC-1 Dual-platform mitochondrial membrane potential analysis

HaCaT cells (1 × 10^5^ cells/well) were seeded in confocal dishes and incubated overnight for adherence. Following 24-h Cu-NZs exposure, cells were loaded with JC-1 working solution (Beyotime) in complete medium at 37 °C/5 % CO_2_ for 30 min under light-protected conditions. For confocal microscopy, live cells were washed with pre-warmed PBS and immediately imaged using Zeiss LSM980. JC-1 monomers were detected at 488 nm excitation/530 ± 30 nm emission (pseudocolored green), while J-aggregates were captured at 561 nm excitation/590 ± 50 nm emission (pseudocolored red). Fluorescence intensity ratios (red:green) were quantified per cell using Gen5 software. For flow cytometric analysis, parallel samples were trypsinized and analyzed on a BD LSRFortessa™, with JC-1 monomers detected in FITC channel (488 nm ex/530 ± 15 nm) and aggregates in PE channel (488 nm ex/575 ± 13 nm). Data were analyzed in FlowJo v10.4.

### 2.17 Transmission electron microscopy (TEM) sample processing

HaCaT cells (1 × 10^7^ cells/100 mm dish) were cultured overnight and treated with Cu-NZs for 24 h. Primary fixation was performed with 2.5 % glutaraldehyde (5 min, RT), followed by gentle unidirectional scraping. Cell suspensions were centrifuged (400×*g*, 5 min) to form pellets, which were subsequently transferred to Wuhan Baichuandu Biotechnology Co., Ltd. For professional processing and TEM imaging.

### 2.18 Intracellular GSH and NADPH analysis

The intracellular levels of GSH and NADPH were quantified using commercial assay kits (Beyotime). HaCaT cells were seeded at a density of 5 × 10^5^ cells per dish and incubated for 24 h, followed by treatment with Cu-NZs at the indicated concentrations for another 24 h. After treatment, cells were harvested, and the content of GSH and NADPH was determined in the supernatant according to the respective manufacturer's instructions. All results were normalized to the control group.

### 2.19 RNA extraction and real-time PCR (RT-qPCR)

Total RNA from murine cutaneous tissues and cultured cells was isolated using MagZol reagent (Magen). For cDNA synthesis, 1 μg of purified RNA underwent reverse transcription with HiScript II Q RT SuperMix (Vazyme Biotech) following manufacturer specifications. Quantitative PCR amplification was performed using ChamQ Blue Universal SYBR qPCR Master Mix (Vazyme Biotech) on an Applied Biosystems QuantStudio 3 Real-Time PCR platform. Relative gene expression quantification employed the comparative ΔΔCt method normalized to GAPDH/β-actin endogenous control. Primer sequences are detailed in Table S4.

### 2.20 Western blotting

Following 24-h Cu-NZs treatment of HaCaT cells (5 × 10^5^/6-well plate), RIPA lysis buffer containing protease/phosphatase inhibitors was applied for 30 min on ice. Lysates were centrifuged (12,000×*g*, 20 min, 4 °C) and supernatants quantified via BCA assay (Jiangsu Cowin Biotech #CW0014S). For DLAT analysis, samples were mixed with non-reducing loading buffer (without heating) and electrophoresed on 8 % SDS-free gels; FDX1 and LIAS samples underwent denaturation in reducing buffer (95 °C, 10 min) followed by electrophoresis on SDS-containing gels (15 % and 10 % respectively). Post-transfer, membranes were blocked with 5 % skim milk (1 h, RT), probed overnight at 4 °C with primary antibodies: DLAT (Proteintech #13426-1-AP, 1:4000), FDX1 (#12592-1-AP, 1:5000), LIAS (#11577-1-AP, 1:2000), GAPDH (#60004-1-Ig, 1:10000), α-Tubulin (#11224-1-AP, 1:5000), washed with TBST, incubated with HRP-conjugated secondaries, and detected using ECL on an Odyssey® Fc system.

### 2.21 Immunofluorescence

HaCaT keratinocytes (5 × 10^4^ cells/confocal dish) were cultured overnight and exposed to Cu-NZs for 24 h. Cells underwent sequential processing: fixation with 4 % paraformaldehyde (15 min, RT), permeabilization with 0.1 % Triton X-100 (10 min), and blocking with universal blocking buffer (ZSGB-BIO, #ZLI-9056; 1 h, RT). Primary antibodies against DLAT (Proteintech, #13426-1-AP) and FDX1 (Proteintech, #12592-1-AP) were diluted (1:200) in antibody diluent (ZSGB-BIO, #ZLI-9029) and applied overnight at 4 °C. Specimens were then incubated with Alexa Fluor® 488-conjugated donkey anti-rabbit IgG (Invitrogen, #A21206; 1:400) for 1 h at RT with light protection. Nuclei were counterstained with DAPI (Servicebio, #G1012; 5 min) prior to mounting. Confocal imaging was performed using a Zeiss LSM system with 63 × oil objective under standardized configurations: DAPI (405 nm excitation/420–480 nm emission), Alexa Fluor 488 (488 nm ex/500–550 nm em). Fluorescence co-localization analysis was conducted in ImageJ v1.53 using background-subtracted intensity measurements.

### 2.22 Cu-NZs-loaded hydrogel construction

A 1 % (w/v) carbomer dispersion was prepared and allowed to fully hydrate. Triethanolamine was added dropwise under gentle agitation to neutralize the polymer and generate a transparent hydrogel matrix. Subsequently, Cu-NZs were introduced at a defined mass ratio and homogeneously dispersed to yield the Cu-NZs-loaded hydrogel.

### 2.23 IMQ-induced psoriasis

Female C57BL/6 mice (8-weeks-old; Hunan SJA Laboratory Animal Co.) received ethical approval from Xiangya Hospital, Central South University. Psoriasiform dermatitis was induced via daily topical application of 5 % imiquimod cream (Sichuan Med-Shine Pharmaceutical) on shaved dorsal skin (6 cm^2^) for 5 consecutive days [36,37]. Therapeutic groups underwent topical administration of Cu-NZs-loaded hydrogel (30 μg Cu^2+^) 6 h post-daily IMQ exposure. Clinical progression was quantified using daily Psoriasis Area and Severity Index (PASI) scoring (0–4 scales for erythema, scaling, and epidermal thickening). On day 5, euthanized mice underwent: 1) Dorsal skin excision for H&E-stained epidermal thickness quantification and qPCR analysis of inflammatory mediators; 2) Systemic toxicological evaluation via histopathology of major organs (heart, liver, spleen, lungs, kidneys), and serum biochemical profiling (ALT/AST/BUN/creatinine).

### 2.24 Immunohistochemistry (IHC)

Skin tissue sections (4 μm) underwent antigen retrieval in EDTA buffer (pH 8.0), followed by blocking and overnight incubation at 4 °C with the following primary antibodies (all from Proteintech): anti-DLAT (#13426-1-AP, 1:100), anti-LIAS (#11577-1-AP, 1:100), and anti-FDX1 (#12592-1-AP, 1:100). Subsequent staining was carried out using an HRP-conjugated secondary antibody and DAB substrate (PV-9000, ZSGB-BIO) according to the manufacturer's protocol. Nuclei were counterstained with hematoxylin. Images were acquired by light microscopy.

### 2.25 Statistical analysis

Data were presented as mean ± SD and were analyzed in GraphPad Prism 10. Group comparisons used: unpaired *t*-test (two groups); one-way ANOVA with Tukey's post-hoc test (≥3 groups). Significance: ∗p < 0.05, ∗∗p < 0.01, ∗∗∗p < 0.001, ∗∗∗∗p < 0.0001; ns: non-significant.

## 3 Results and discussion

### 3.1 Cuproptosis-linked biomarkers were profiled in psoriasis patients using bioinformatic analysis

Based on relevant literature [26,27], we identified 14 genes linked to cuproptosis: FDX1, LIPT1, DLD, LIAS, DLAT, PDHA1, PDHB, MTF1, GLS, CDKN2A, ATP7B, ATP7A, DLST and SLC31A1. Analysis of the public GEO dataset GSE74697 revealed that, relative to healthy controls, all 14 genes were uniformly downregulated in both the cluster heat-map and volcano plot. This starkly contrasts with the upregulated expression of IL-17, IL-23, and S100 family genes in psoriasis patients (Fig. 1b and c). Furthermore, Spearman's correlation analysis revealed significant negative correlations between these cuproptosis-related genes and the IL-17 family (IL17A, IL17F), S100 family (S100A7, S100A8, S100A9), and IL-23 (Fig. 1d). Additionally, we used the Mantel test to evaluate the correlations between the inflammatory gene families and the cuproptosis-related genes described above. The analysis revealed a weak negative correlation between inflammatory gene sets and several cuproptosis-related genes, whereas the cuproptosis genes themselves were positively inter-correlated (Fig. 1e). These data indicated that the down-regulation of cuproptosis genes in psoriasis parallelled the degree of inflammation, providing a rationale for therapeutic cuproptosis induction.

### 3.2 Synthesis and characterization of Cu-NZs

**Fig. 2:**
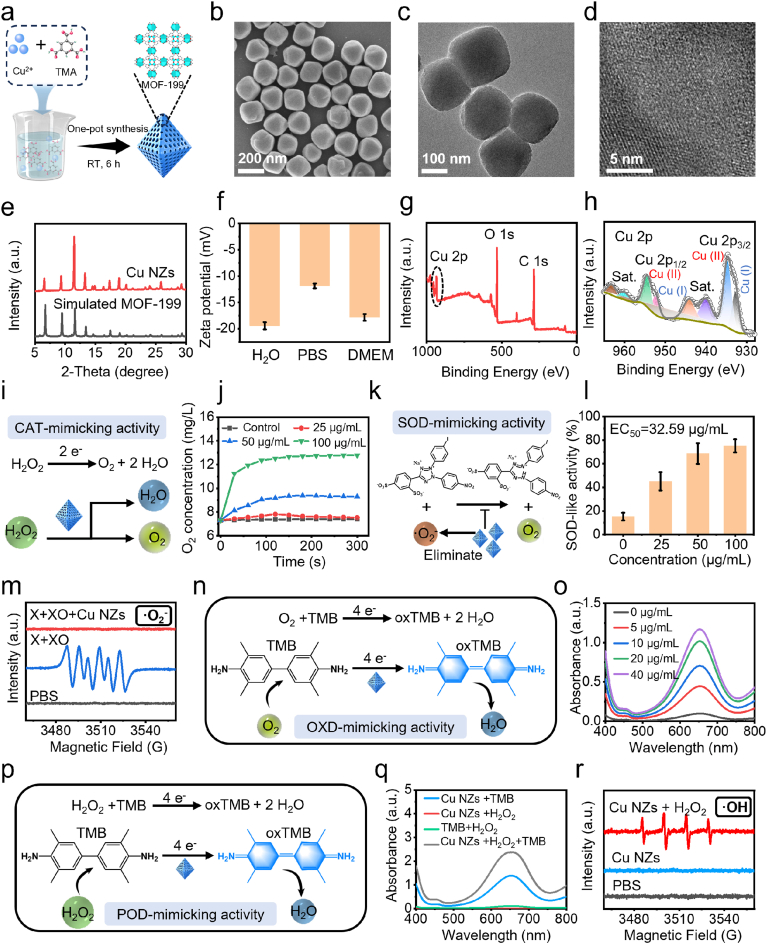
**Characterization of the Cu-NZs.** (a) Synthetic process of Cu-NZs. (b) SEM image, scale bar, 200 nm, and (c) TEM image of Cu-NZs, scale bar, 100 nm. (d) HR-TEM of Cu-NZs. Scale bar, 5 nm. (e) PXRD pattern of Cu-NZs. (f) Zeta potential of Cu-NZs in different solvents. Mean ± SD, n = 3. (g) Full XPS scanning spectra of the Cu-NZs. (h) XPS Cu 2p spectra of Cu-NZs. (i) Schematic illustration of the CAT-mimicking activity mechanism of Cu-NZs. (j) Kinetic curves of O_2_ generation from the decomposition of H_2_O_2_ (50 mM) in the presence of different concentrations of Cu-NZs. (k) Schematic illustration of the SOD-mimicking activity mechanism of Cu-NZs. (l) Elimination efficiency of •O_2_- with different concentrations of Cu-NZs. Mean ± SD, n = 3. (m) ESR spectra of •O_2_- trapped by TEMP after different treatments. (n) Schematic illustration of the OXD-mimicking activity mechanism of Cu-NZs. (o) OXD-mimicking activity in the presence of different concentrations of Cu-NZs. Mean ± SD, n = 3. (p) Schematic illustration of the POD-mimicking activity mechanism of Cu-NZs. (q) The POD-like activity of Cu-NZs. Mean ± SD, n = 3. (r) ESR spectra of •OH trapped by DMPO after different treatments.

Cu-NZs were synthesized via a one-step method according to a previous report with some modifications by adding PVP-K30 to change the size of Cu-NZs [38] (Fig. 2a). SEM (Fig. 2b) and TEM (Fig. 2c) images revealed octahedral or domorphology of Cu-NZs with an average diameter of 165 ± 18.7 nm (Fig. S1). A high-resolution TEM (HRTEM) image revealed the crystalline characteristics of Cu-NZs with a lattice fringe (Fig. 2d). Elemental mapping images confirmed the existence of Cu, C, and O elements within the nanoparticles (Fig. S2). The X-ray diffraction (XRD) pattern of Cu-NZs indicated the well-developed crystalline matched well with the simulated pattern of Cu-NZs [38] (Fig. 2e). The zeta potentials of Cu-NZs suspended in different mediums were determined. According to the dynamic light scattering (DLS) measurement, the Cu-NZs retained hydrodynamic diameters of 157.23 nm, 144.58 nm, and 177.89 nm in water, PBS, and DMEM, respectively (Fig. S3), with corresponding ζ-potentials of −19.4, −11.8, and −17.8 mV, confirming their colloidal stability under physiological conditions (Fig. 2f). The zeta potentials of Cu-NZs suspended in water, PBS, and DMEM were −19.4 mV, −11.8 mV, and −17.8 mV, respectively, suggesting Cu-NZs were physiologically stable in these mediums (Fig. 2f). Thermogravimetric analysis (TGA) of the Cu-NZs revealed that the assembled TMA constitutes 43.2 % of the total mass (Fig. S3), while the concentration of Cu species was 1950 μg/mL assayed by inductively coupled plasma (ICP-MS). Fourier-transform infrared (FTIR) spectra revealed the stretching vibration of O-H (3423 cm^−1^), phenyl ring (1587, and 1448 cm^−1^), and Cu-O (493 cm^−1^), assigned to Cu-NZs, respectively (Fig. S4).

The X-ray photoelectron spectroscopy (XPS) spectra further elucidated the chemical composition, showing the presence of Cu 2p (934.3 eV, 27.54 %), C 1s (285.2 eV, 65.70 %) and O 1s (532.0 eV, 27.54 %) for (Fig. 2g–Table S1). The high-resolution Cu 2p spectrum showed two characteristic peaks at 934.6 eV (Cu 2p_3/2_) and 954.2 eV (Cu 2p_1/2_) accompanied by two shake-up satellite peaks, which were the typical values for Cu^2+^ [39,40] (Fig. 2h). Furthermore, Cu LMM Auger electron spectroscopy demonstrated that the peak at 944.1 eV and 932.5 eV, was assigned to Cu^+^ [41]. Based on the integrated peak area ratio, the percentages of Cu^+^ and Cu^2+^ were estimated to be 33.25 % and 66.75 %, respectively (Table S2). Meanwhile, the O 1s spectrum exhibited three characteristic peaks, assigning the oxygen configuration in O-C (533.2 eV), O=C (531.6 eV), and O-Cu (530.0 eV), respectively (Fig. S5). In addition, the C 1s signals confirmed the presence of Cu-NZs in C-C/C=C (288.5 eV), C-O (286.2 eV), and O-C=O (284.8 eV), respectively (Fig. S6, Table S3). All these findings collectively confirmed the successful preparation of Cu-NZs.

### 3.3 Multienzyme activities of Cu-NZs

The catalytic ability of Cu-NZs was systematically explored through a series of enzyme-mimetic activity assays. The catalase (CAT) mimics H_2_O_2_ of Cu-NZs were evaluated by monitoring the concentration of O_2_ dissolved in H_2_O_2_ (50 mM) solutions containing varied concentrations of Cu-NZs [42] (Fig. 2i). It is shown that the Cu-NZs could efficiently catalyze the decomposition of H_2_O_2_. Meanwhile, the production of O_2_ increased from 7.3 mg/L to 12.8 mg/L as Cu-NZs increased from 0 μg/mL to 100 μg/mL (Fig. 2j, Fig. S7). Digital photographs taken in 2 h of the oxygenation reaction revealed that a large number of bubbles were generated for the assay at 100 μg/mL of Cu-NZs (Fig. S8). The superoxide dismutase (SOD)-like activity of Cu-NZs was evaluated via colorimetric analysis [41] (Fig. 2k). In the superoxide dismutation assay, SOD-like activity rose with Cu-NZ dose, reaching 75.2 % at 100 μg/mL (Fig. 2l). Through logistic function fitting, we showed that a Cu-NZs dose of 32.59 μg/mL was needed to reach 50 % of relative SOD-like activity (Fig. S9). These results confirmed that the absorbance decrease reflects genuine SOD-like activity of the Cu-NZs. Furthermore, the ESR spectra was used to further validate the SOD-like activity of Cu-NZs. After the co-incubation of xanthine (X) with xanthine oxidase (XO), characteristic peaks of •O_2_^−^ appeared. As expected, Cu-NZs effectively scavenged •O_2_^−^, as demonstrated by the reduction of the EPR amplitude of the peak of DMPO−OOH (Fig. 2m).

Next, oxidase (OXD)-like activity was examined, during which O_2_ was reduced to water. In a typical OXD-like catalytic reaction, a chromogenic substrate, 3,3′,5,5′-tetramethylbenzidine (TMB) with a blue product of oxidized TMB (oxTMB) that exhibited characteristic absorbance at 650 nm [43] (Fig. 2n). A considerable increased in the absorption intensity at 650 nm was detected with an increase in Cu-NZs concentration, confirming its OXD-like activity (Fig. 2o). In addition, the absorbance of oxTMB enhanced with increasing TMB concentration. (Fig. S10). Peroxidase (POD) naturally converted H_2_O_2_ to H_2_O while generating •OH. In the presence of H_2_O_2_, Cu-NZs catalyzed the oxidation of colorless TMB to blue oxTMB, producing an absorption peak at 650 nm that indirectly evidences •OH formation (Fig. 2p, Fig. S11). Moreover, the absorbance of oxTMB at 650 nm rose markedly only when Cu-NZs, H_2_O_2_ and TMB were present together, indicating amplified •OH production; the peak remained negligible in the Cu-NZs + TMB or TMB + H_2_O_2_ controls (Fig. 2q, Fig. S12). Further, the TMB oxidation level was remarkably affected by adjusting the different concentrations of TMB, H_2_O_2_, and Cu-NZs (Figs. S13 and S14). These results indicated that Cu-NZs showed the high POD-like activity. The generation of free •OH radicals was subsequently verified by ESR spectroscopy with 5,5-dimethyl-1-pyrroline N-oxide (DMPO) as the spin trap [44]. A specific ESR signal with an integral intensity of 1:2:2:1 was clearly observed in the copresence of Cu-NZs + H_2_O_2_. No interpretable ESR signals were detected when DMPO was mixed with Cu-NZs or H_2_O_2_ (Fig. 2r). These combined attributes made Cu-NZs highly efficient ROS generators via enzymatic cascade amplification, thereby potentiated cuproptosis-driven therapy.

### 3.4 Catalytic mechanism revealed by density functional theory calculation

**Fig. 3:**
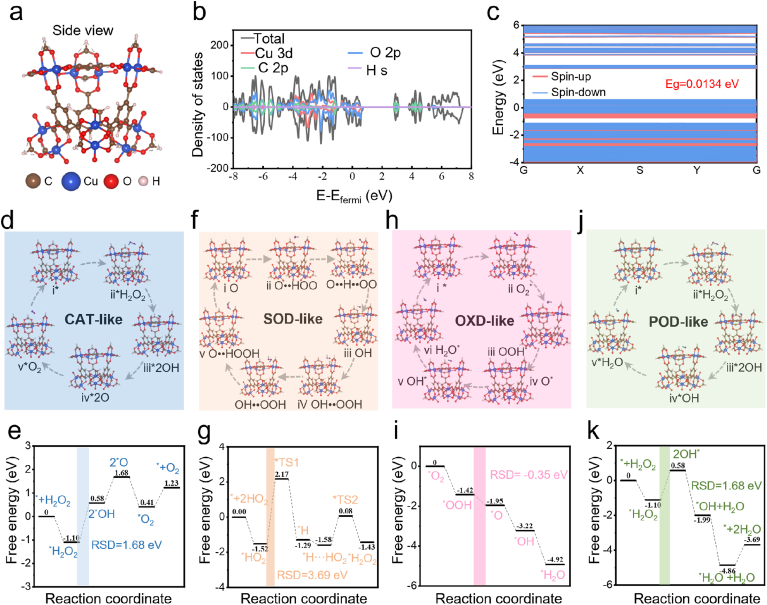
**DFT explorations on the CAT-, SOD-, OXD-, and POD-mimicking activities of Cu-NZs.** (a) Polyhedral view of Cu-NZs with periodic boundary conditions (PBC). Brown, blue, red, and pink balls illustrate C, Cu, O, and H atoms. (b) Electronic band structure and (c) density of states of Cu-NZs. (d) CAT-like reaction, (f) SOD-like reaction, (h) OXD-like reaction and (j) POD-like reaction for Cu-NZs. Corresponding free energy diagrams of (e) CAT-like reaction, (g) SOD-like reaction, (i) OXD-like reaction, and (k) POD-like reaction for Cu-NZs.

All the density functional theory (DFT) calculations were conducted based on first principles implemented in the Vienna Ab-inito Simulation Package (VASP) to predict Cu-NZs multienzyme-like activities and reveal the underlying mechanism via energy and electronic structure calculations [45]. A periodic structure model of Cu-NZs was constructed to simulate the multienzyme activities (Fig. 3a). Next, the electronic band structure and density of states were calculated after geometric optimization. The energy band structure indicated the excellent electrical conductivity of Cu-NZs as several bands crossed the Fermi level, which was advantageous for catalytic reactions involving electron transfer (Fig. 3b and c). In addition, the density of states (DOS) near the Fermi level was predominantly contributed by Cu 3d and O 2p orbitals, indicating that electrons near the Fermi level were significantly located on the Cu atoms, which played a crucial role in the catalytic activity of Cu-NZs. The unsaturated d orbitals of the Cu metal in the Cu-NZs allowed for easy interaction with the reactant molecules, facilitating the electron transfer process. These results enabled Cu-NZs with the excellent catalytic performance of activating H_2_O_2_ in CAT-like and POD-like reactions, desorbing H_2_O in OXD-like and POD-like reactions to recover catalytic active surface, •O_2_^−^ desorption in SOD-like reaction and O_2_ desorption in CAT-like reaction, respectively.

The reaction pathways and energy profiles for the CAT-like activity of Cu-NZs were illustrated in Fig. 3d. In the proposed mechanism, one H_2_O_2_ molecule was initially adsorbed onto the surface of Cu-NZs, which then uniformly dissociated 2 OH∗ species on the Cu-O clusters. Subsequently, 2 OH∗ were dehydrogenated into 2O∗ species, which was the rate-determining step (RDS) for Cu-NZs in CAT-like reaction. The 2O∗ species combine into O_2_∗ and eventually desorbed to regenerate active sites (Fig. 3d). As shown in Fig. 3e, the CAT-like reaction on Cu-NZs was more thermodynamically favorable. The energy increase for this step was 1.68 eV, identifying it as the rate-determining step. These results confirmed the CAT-mimicking characteristics of Cu-NZs, facilitating the catalytic the H_2_O_2_ to O_2_.

The SOD-like catalytic cycle of a catalytic site consisted of two elementary reactions including an oxidation reaction and a reduction reaction. A carbonyl group of Cu-O clusters could be converted to a hydroxyl group by oxidizing a ∗HO_2_ free radical to produce an O_2_ molecule, and a hydroxyl group of Cu-O clusters could be converted to a carbonyl group by reducing a ∗HO_2_ free radical to produce an H_2_O_2_ molecule (Fig. 3f). Fig. 3g indicated that there were both seven different states including two transition states (named TS1/TS2) in the proposed reaction pathway for the Cu-O cluster of Cu-NZs to achieve the SOD-like catalytic cycle. The ate-determining step was 3.69 eV. These results confirmed the SOD-mimicking characteristics of Cu-NZs, facilitating the catalytic the •O_2_^−^ to H_2_O_2_ and O_2_.

For the OXD-like reaction, the proposed reaction pathways and calculated energy profiles were shown in Fig. 3h and i. The OXD-like reaction initiated with the adsorption and subsequent hydrogenation of O_2_ on the Cu-NZs surface to form ∗OOH species; the adsorption energy for this step was −1.42 eV. Simultaneously, the ∗O species reacted with an approaching H^+^ to form surface-adsorbed ∗OH, releasing 1.27 eV of energy. This adsorbed ∗OH then underwent further hydrogenation on the Cu-NZs to yield an H_2_O molecule. These one step resulted in energy losses of 1.7 eV. The ate-determining step was −0.35 eV. Cu-NZs participated in a new catalytic cycle after the release of H_2_O molecules, confirming the XOD-mimicking characteristics of Cu-NZs.

The proposed reaction pathways and calculated energy profiles for the POD-like activity of Cu-NZs were illustrated in Fig. 3j and k. It was demonstrated that H_2_O_2_ was captured by the Cu-NZs and adsorbed on Cu-O clusters with an adsorption energy of −1.10 eV, indicating that the binding of H_2_O_2_ to the Cu-NZs was energetically favorable. Subsequently, it decomposed into two OH∗ ions with an energy drop of 0.58 eV. Subsequently, an increase in energy (1.68 eV) occurred as a result of the release of OH∗, which was the rate-determining step. Simultaneously, the other OH∗ species reacted with the approaching H^+^ to form H_2_O adsorbed on the surface of Cu-NZs, with an energy drop of 2.57 eV. Finally, Cu-NZs recovered with an energy increase of 1.17 eV following the formation of the products. The above results demonstrated that the multi-enzyme mimicking activities of Cu-NZs has been theoretically verified through a detailed investigation of the proposed reaction pathways and energy profiles. Thus, Cu-NZs acted as stable multi-enzyme catalysts in the neutral superficial horizon, where they markedly amplified ROS generation through an enzymatic cascade; this heightened oxidative burst subsequently potentiated cuproptosis-driven therapy once the particles reach the acidic, copper-releasing niche.

### 3.5 Cu-NZs suppressed HaCaT cell proliferation and induce mitochondrial damage in vitro

**Fig. 4:**
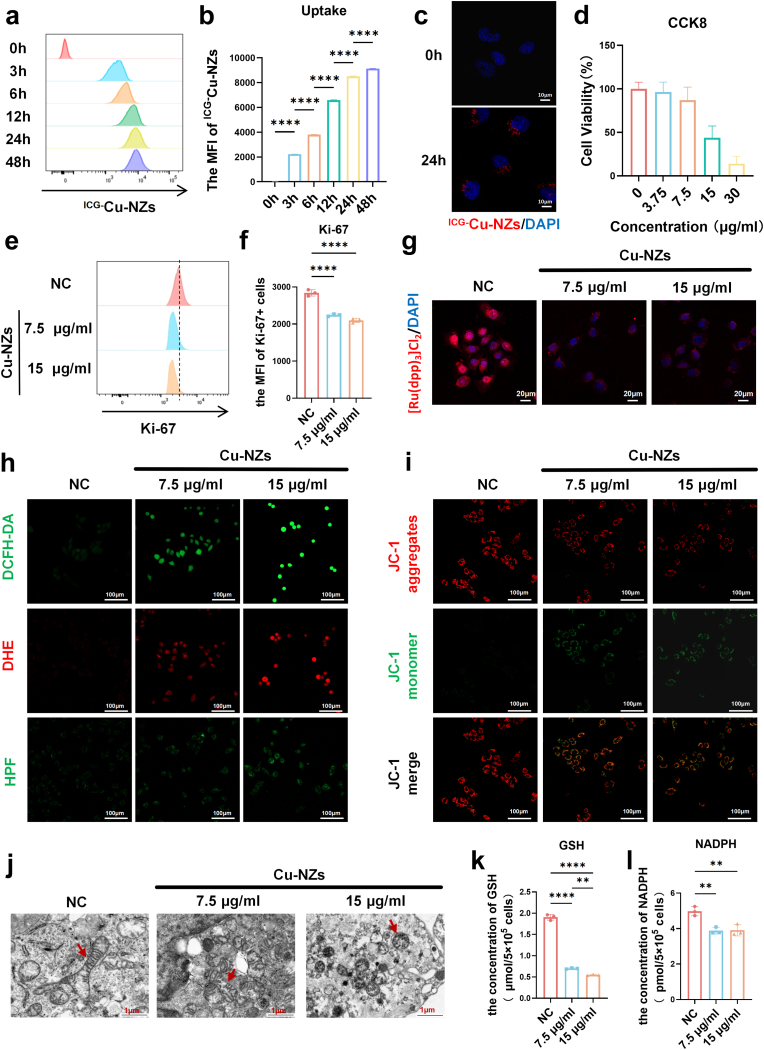
**In vitro cellular uptake, cytotoxicity, proliferation, and mitochondrial function of Cu-NZs.** (a) Cellular uptake of ^ICG−^Cu-NZs (5 μg/mL) in HaCaT cells at various time points (0, 3, 6, 12, 24, 48 h). (b) Quantification of mean fluorescence intensity (MFI) from (a). (c) Confocal microscopy images showing Cellular uptake of ^ICG−^Cu-NZs (5 μg/mL) in HaCaT cells. (d) Cytotoxicity of HaCaT cells treated with increasing Cu-NZs concentrations (0–30 μg/mL) for 24 h. (e,f) Flow cytometry analysis and quantification of Ki-67 expression in HaCaT cells treated with Cu-NZs (7.5 and 15 μg/mL) for 24 h. (g) Confocal microscopy images showing the determination of intracellular oxygenation in HaCaT cells using the [Ru(dpp)_3_]Cl_2_ oxygen probe. (h) Confocal microscopy images showing the expression of ROS in HaCaT cells treated with Cu-NZs (7.5 and 15 μg/mL) for 24 h, as detected by DCFH-DA, DHE and HPF probes. (i) Confocal microscopy images showing JC-1 monomer/polymer shift (indicating mitochondrial membrane potential changes) in HaCaT cells treated with Cu-NZs (7.5 and 15 μg/mL) for 24 h. (j) TEM images showing mitochondrial morphology changes in HaCaT cells treated with Cu-NZs (7.5 and 15 μg/mL) for 24 h. (k) Measurement of GSH levels in HaCaT cells with Cu-NZs (7.5 and 15 μg/mL) for 24 h. (l) Measurement of NADPH levels in HaCaT cells with Cu-NZs (7.5 and 15 μg/mL) for 24 h. All data are presented as mean ± SD (n = 3). Group comparisons were analyzed using one-way ANOVA with Tukey's post hoc test. Statistical significance is indicated as follows: ∗∗p < 0.01, ∗∗∗∗p < 0.0001.

To investigate the effects of Cu-NZs on human keratinocytes, we employed the HaCaT cell line for in vitro interventions in our cellular experiments. HaCaT is an immortalized cell line derived from normal human skin keratinocytes and is widely used in dermatological research and toxicological assessments. To evaluate the cellular uptake of Cu-NZs, we labeled it with indocyanine green (ICG). Time-dependent internalization by HaCaT cells was observed, reaching near-maximal levels at 24 h (Fig. 4a and b). This uptake was further visualized qualitatively by confocal microscopy at the 24-h time point (Fig. 4c). Consequently, all subsequent in vitro experiments utilized this 24-h timepoint. To assess Cu-NZs cytotoxicity in HaCaT cells, a CCK-8 assay was conducted. This colorimetric method measures cell viability and proliferation by quantifying the absorbance of the reduced formazan product generated by cellular dehydrogenases. Results indicated approximately 50 % cell survival at 15 μg/mL Cu-NZs (Fig. 4d). Based on this, concentrations of 7.5 μg/mL and 15 μg/mL were selected for further studies. We further investigated the impact of Cu-NZs on HaCaT cell proliferation using Ki-67, a nuclear protein biomarker widely used to assess proliferative activity. Flow cytometry analysis of Ki-67 expression revealed that 24-h Cu-NZs exposure induced significant anti-proliferative effects compared to the control group (Fig. 4e and f). Given the multi-enzyme activity of Cu-NZs and their potential to release O_2_ and alleviate hypoxia, we next examined intracellular oxygen levels and hypoxia status after treatment. Intracellular oxygen was detected using the oxygen-sensitive probe [Ru(dpp)_3_]Cl_2_, whose fluorescence is quenched upon binding to O_2_. Concurrently, transcriptional expression of hif1α, a key hypoxia-related transcription factor, was measured by qPCR. The results showed that Cu-NZs treatment significantly quenched [Ru(dpp)_3_]Cl_2_ fluorescence (Fig. 4g) and downregulated hif1α mRNA (Fig. S16), indicating enhanced intracellular oxygenation and attenuated hypoxia. Given that the enzymatic generation of O_2_ by Cu-NZs could potentially lead to an overload of intracellular ROS, and based on established evidence that intracellular copper ions disrupt mitochondrial respiratory chain function across cell types [46], we next investigated the impact of Cu-NZs on mitochondrial status. Treatment with Cu-NZs significantly elevated intracellular ROS levels, as detected by multiple fluorescent probes (DCFH-DA, DHE, and HPF) (Fig. 4h–S17). Subsequently, we assessed mitochondrial membrane potential (ΔΨm) using JC-1, a fluorescent probe that exhibits a spectral shift (red → green) upon ΔΨm dissipation, serving as an early indicator of apoptosis. JC-1 staining demonstrated that Cu-NZs significantly altered ΔΨm in HaCaT cells (Fig. 4i–S18). To characterize ultrastructural changes in mitochondria following Cu-NZs internalization, we performed TEM, a technique enabling atomic-scale resolution (0.1–0.2 nm) for direct visualization of cellular and material microstructure. TEM revealed Cu-NZs-induced mitochondrial damage compared to controls, characterized by loss of normal morphology, reduced size, rounding, and increased electron density of cristae (Fig. 4j). Following the above findings that Cu-NZs significantly increased intracellular ROS levels, we further examined the core antioxidant system to elucidate the underlying mechanism. The results indicated that Cu-NZs treatment not only induced ROS generation but also concurrently depleted key intracellular antioxidant molecules. GSH, the primary antioxidant responsible for directly scavenging ROS, showed a significant decrease in its levels (Fig. 4k). Meanwhile, NADPH, which supplies the reducing power for GSH regeneration, was also markedly reduced (Fig. 4l). The simultaneous depletion of both GSH and NADPH indicated that the cellular antioxidant defense capacity was severely compromised, which directly led to impaired ROS clearance and sustained accumulation, thereby exacerbating oxidative stress. Interestingly, our findings also showed that Cu-NZs significantly suppressed inflammatory cytokine expression in M5-stimulated HaCaT cells (an in vitro model mimicking psoriatic inflammatory pathology) (Fig. S19), while ROS are known to promote inflammatory cytokine expression (including IL-1β, IL-6, and IL-8) via activation pathways [47,48]. Based on these results, we speculated that ROS may function as a double-edged sword. Beyond a certain threshold, the substantial ROS burst induced by Cu-NZs, coupled with the depletion of antioxidant reserved (GSH/NADPH), might create an overwhelmingly pro-oxidant environment. This extreme oxidative stress could activate cellular damage-control programs that broadly suppressed energy-intensive processes like inflammatory cytokine production, or specifically inhibit the activation of inflammasomes and other ROS-sensitive inflammatory complexes. Collectively, these results demonstrated that Cu-NZs exerted their effects not only by significantly inhibiting the in vitro proliferation of HaCaT cells and elevating intracellular ROS levels to induce mitochondrial impairment, but also by suppressing the expression of key inflammatory cytokines as a consequential or supporting mechanism.

### 3.6 Cu-NZs inhibited HaCaT cell proliferation by inducing cuproptosis

**Fig. 5:**
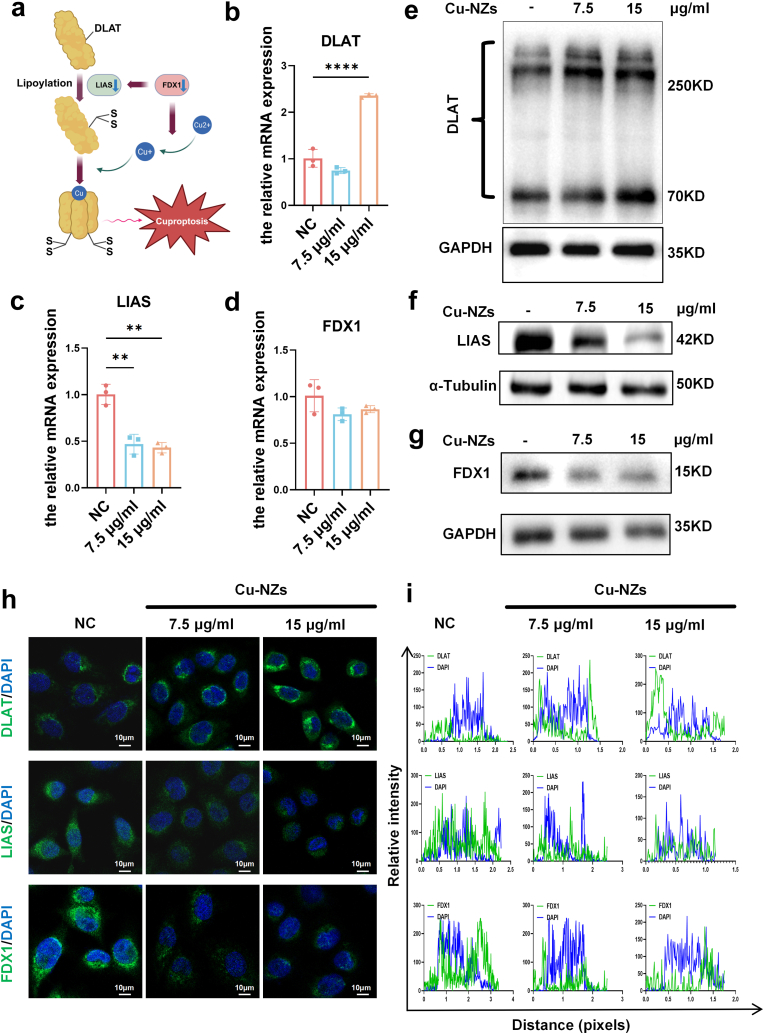
**Studies on cuproptosis mediated by Cu-NZs in vitro.** (a) Schematic illustration of the cuproptosis pathway. (b–d) Relative mRNA expression levels of cuproptosis-related genes (DLAT, FDX1, LIAS) in HaCaT cells treated with Cu-NZs (7.5 and 15 μg/mL) for 24 h. (e–g) Western blot analysis of cuproptosis-related proteins (DLAT, FDX1, LIAS) in HaCaT cells treated with Cu-NZs (7.5 and 15 μg/mL) for 24 h. (h,i) Confocal microscopy images showing expression and subcellular localization changes of cuproptosis-related proteins (DLAT, LIAS and FDX1) in HaCaT cells treated with Cu-NZs (7.5 and 15 μg/mL) for 24 h (representative images shown). All data are presented as mean ± SD (n = 3). Statistical comparisons were performed using one-way ANOVA followed by Tukey's post hoc test. Significance levels are indicated as follows: ∗∗p < 0.01, ∗∗∗p < 0.001, and ∗∗∗∗p < 0.0001.

In the above results, we found that Cu-NZs inhibited HaCaT cell proliferation and disrupt mitochondrial function. Previous literature has reported a Cu^2+^-dependent cell death modality—cuproptosis—characterized by Cu^2+^ entry into mitochondria promoting the formation of disulfide-linked oligomers of DLAT [11]. In the specific biological process, FDX1 donates electrons to LIAS for lipoic acid synthesis; the newly formed lipoate is then covalently attached to DLAT, generating lipoylated DLAT dimers that are required for pyruvate-dehydrogenase-complex assembly and activity. During cuproptosis this FDX1→LIAS→DLAT lipoylation axis is hyper-activated, causing pathological aggregation of the lipoylated DLAT dimers and consequent cell death (Fig. 5a). To determine whether Cu-NZs suppress HaCaT proliferation by eliciting cuproptosis, we performed RT-qPCR. Cells treated with 15 μg/mL Cu-NZs significantly elevated DLAT mRNA compare with the control group cells (Fig. 5b), while LIAS was significantly downregulated at 7.5 μg/ml and 15 μg/ml (Fig. 5c), and FDX1 also showed a downregulation trend, although without statistical significance (Fig. 5d). Subsequently, Western blot further proved that DLAT oligomers were significantly increased after Cu-NZs treatment (Fig. 5e), while FDX1 and LIAS showed significantly decreasing trends (Fig. 5f and g). Immunofluorescence confirmed the formation of DLAT oligomers and reduced expression of LIAS and FDX1 (Fig. 5h), which was further supported by their subcellular co-localization analysis (Fig. 5i). Collectively, these data demonstrated that Cu-NZs suppress HaCaT proliferation by inducing cuproptosis, underscoring their therapeutic potential against psoriasis.

### 3.7 Topical Cu-NZs gel ameliorated Psoriasiform dermatitis in IMQ-induced mice

**Fig. 6:**
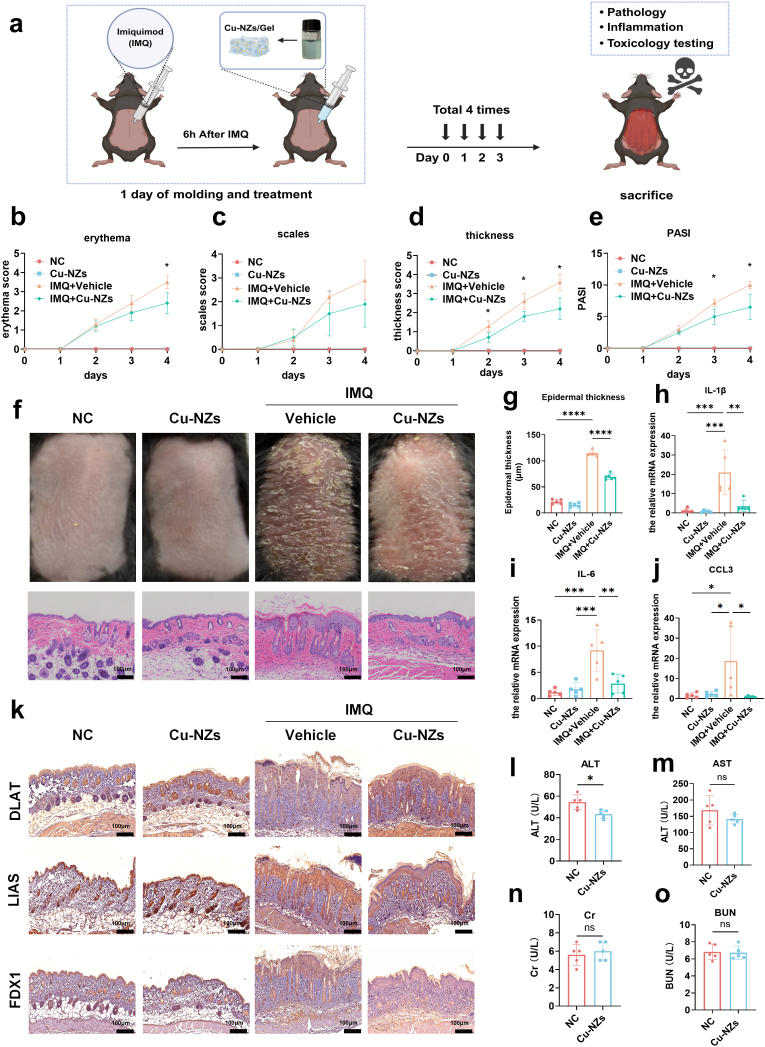
**In vivo alleviation of psoriatic-like lesions by Cu-NZs in C57 mice.** (a) Experimental schedule of the therapeutic regimen in IMQ-induced psoriatic mouse models. (b–e) Psoriasis Area and Severity Index (PASI) scores assessing erythema, scaling, and skin thickness. (f) Macroscopic skin appearance and corresponding H&E-stained skin sections. (g) Epidermal thickness measurements from H&E-stained sections. (h–j) mRNA expression levels of inflammatory cytokines in skin tissues. (k) Expression of cuproptosis-related proteins (DLAT, LIAS, FDX1) in mouse skin as detected by IHC. (l–o) Liver and kidney function markers: alanine aminotransferase (ALT), aspartate aminotransferase (AST), serum creatinine (SCr), and blood urea nitrogen (BUN). All data are presented as mean ± SD (n = 5 per group). For comparisons between two groups, an unpaired *t*-test was used. For comparisons involving three or more groups, one-way ANOVA followed by Tukey's post hoc test was performed. Significance levels are indicated as follows: ns, not significant; ∗p < 0.05; ∗∗p < 0.01; ∗∗∗p < 0.001; ∗∗∗∗p < 0.0001.

Having shown that Cu-NZs suppressed HaCaT proliferation and inflammation by boosting ROS and activating the cuproptosis machinery, we next evaluated their therapeutic potential. To validate the therapeutic efficacy of Cu-NZs against psoriasis, we made a Carbomer-based Cu-NZs/gel and applied it topically to IMQ-induced psoriatic mice throughout the modeling period (Fig. S15). The microscopic internal structure of Cu-NZs/gel was further elucidated by analyzing SEM. As shown in Fig. S15a, the nanoparticles within the freeze-dried Cu-NZs/gel intersected at the joints, forming an intricate interpenetrating network within the hydrogel, which was different form structure of free gel (Fig. S15b). The energy dispersive spectroscopy elemental mappings revealed a uniform distribution of C, O, and Cu elements throughout the Cu-NZs/gel (Fig. S15c). As the Cu elements originated from Cu-NZs, the results indicated that the gels were chemically homogeneous. The collective findings proved the crosslinking between Cu-NZs with hydrogel and the successful construction of high-performance Cu-NZs/gel. After modeling completion, we collected skin tissues for histopathological staining while assessing drug toxicity and biodistribution in mouse organs (Fig. 6a). Throughout modeling and drug administration, we evaluated mice using the Psoriasis Area and Severity Index (PASI). This revealed that skin erythema, scaling, and thickness progressively worsened over time in the model group, whereas these phenotypes were significantly alleviated in the Cu-NZs-treated group (Fig. 6b, c, d). Compared to the model group, the Cu-NZs group also showed a significantly reduced PASI score (Fig. 6e). H&E staining of central lesional skin demonstrated epidermal hyperplasia (Fig. 6f), with results indicating significantly reduced epidermal thickness in the Cu-NZs group versus the model group (Fig. 6g). To determine whether Cu-NZs ameliorate inflammation in the skin tissue of IMQ-treated mice, we further examined the expression of inflammatory factors at the RNA level. The analysis showed that Cu-NZs attenuated the IMQ-induced upregulation of cytokines, including IL-1β, IL-6, and CCL3 (Fig. 6h, i, j). This corroborated its ability to mitigate IMQ-induced psoriatic phenotypes in mice. To further investigate whether Cu-NZs alleviated psoriasis in vivo by activating the cuproptosis pathway, we performed immunohistochemical staining of key cuproptosis-related markers (DLAT, LIAS, and FDX1) on skin tissues from the mouse model. The results showed that topical treatment with Cu-NZs gel significantly increased DLAT expression while decreasing LIAS and FDX1 levels in keratinocytes (Fig. 6k). These in vivo findings were consistent with our cellular data, collectively supporting the role of cuproptosis in the therapeutic mechanism of Cu-NZs. Finally, to determine whether percutaneous absorption of Cu-NZs caused residual accumulation in organs or induced damage, we assessed liver and kidney function markers (alanine aminotransferase, aspartate aminotransferase, serum creatinine, and blood urea nitrogen). Results showed no elevation in these parameters following Cu-NZs treatment (Fig. 6l, m, n, o). H&E staining of organs (liver, heart, spleen, lungs, kidneys) also revealed no significant abnormalities (Fig. S20). Thus, these results indicated that topical Cu-NZ gel markedly attenuated IMQ-induced psoriasis in mice, reducing PASI scores, epidermal thickness and cutaneous inflammatory cytokines without inflicting organ toxicity. These data established both efficacy and safety, providing a pre-clinical basis for translating Cu-NZs to clinical use.

Extending the tumor-proven “acid-unlocked Cu-ion-cuproptosis” strategy, our multi-enzyme (SOD-CAT-OXD-POD) Cu-nanozyme self-amplifies ROS and trigger keratinocyte cuproptosis at low dose copper, translating the paradigm from cancer therapy to psoriasis [[49], [50], [51], [52], [53], [54]].

## 4 Conclusion

In summary, we proved cuproptosis induction as a novel therapeutic strategy for psoriasis treatment, then developed Cu-NZs that synergistically integrated multi-enzymatic cascades to amplify cuproptosis in keratinocytes for psoriasis treatment. Systematic characterization revealed that the Cu-NZs possessed intrinsic CAT-, SOD-, OXD-, and POD-like enzymatic activities; through multi-enzymatic cascades, it generated ROS, relieved hypoxia, depleted endogenous antioxidant/copper-chelating pools such as GSH and NADPH, and ultimately triggered augmented cuproptosis. Upon skin penetration and uptake by keratinocytes, the nanozymes generated a burst of ROS that collapsed mitochondrial fitness, evoking cuproptosis characterized by the loss of Fe-S cluster proteins and aggregation of lipoylated mitochondrial enzymes. In vivo results, this cascade catalysis underpinned the striking therapeutic index of Cu-NZs, which markedly attenuated IMQ-induced psoriatic phenotypes in mice without detectable systemic or organ toxicity, underscoring the clinical potential of this copper-based multi-enzymatic modality. While the present study has established cuproptosis as a tractable axis for psoriatic inflammation, several limitations must be acknowledged: (i) The IMQ mouse model recapitulates only the acute phase of psoriasis; validation in chronic models is needed to probe long-term efficacy and potential relapse. (ii) Human plaques are 3- to 4-fold thicker than murine skin; incorporation of microneedle or ultrasound-mediated follicular delivery should be explored to ensure uniform nano-enzyme deposition across the full depth of human stratum corneum. Addressing these translational gaps will position copper-catalytic cuproptosis as a viable, safety-locked modality for chronic plaque psoriasis. Overall, This work established a paradigm for providing new perspectives multi-nanozyme catalysis to enhance cuproptosis for developing psoriasis therapies.

## CRediT authorship contribution statement

**Junyu Zhou:** Conceptualization, Data curation, Formal analysis, Methodology, Writing – original draft. **Nianzhou Yu:** Data curation, Methodology. **Xiaoxin Yang:** Funding acquisition, Writing – review & editing. **Shanghong Li:** Methodology, Writing – review & editing. **Mi Huang:** Writing – review & editing. **Tianyi Pang:** Writing – review & editing. **Dong Zhong:** Writing – review & editing. **Yu Wen:** Funding acquisition, Writing – original draft, Writing – review & editing. **Hong Liu:** Conceptualization, Funding acquisition, Writing – review & editing.

## Declaration of competing interest

The authors declare that they have no known competing financial interests or personal relationships that could have appeared to influence the work reported in this paper.

## Data Availability

Data will be made available on request.

## References

[1] Griffiths C.E.M., Armstrong A.W., Gudjonsson J.E., Barker J.N.W.N. (2021). Psoriasis. Lancet.

[2] Armstrong A.W., Blauvelt A., Callis Duffin K., Huang Y.-H., Savage L.J., Guo L., Merola J.F. (2025). Psoriasis. Nat. Rev. Dis. Primers.

[3] Prema S.S., Shanmugamprema D. (2025). Systemic psoriasis: from molecular mechanisms to global management strategies. Clin. Rev. Allergy Immunol..

[4] Xu L., Shao Z., Fang X., Xin Z., Zhao S., Zhang H., Zhang Y., Zheng W., Yu X., Zhang Z., Sun L. (2025). Exploring precision treatments in immune-mediated inflammatory diseases: harnessing the infinite potential of nucleic acid delivery. Explorations.

[5] Wroński A., Wójcik P. (2022). Impact of ROS-dependent lipid metabolism on psoriasis pathophysiology. Int. J. Mol. Sci..

[6] Hong D., Xiong H., Lu S., Ma J., Shi Z. (2025). Metabolic regulation of the immune cell in psoriasis: mechanisms and interventions. Curr. Opin. Immunol..

[7] Long F., Wei X., Chen Y., Li M., Lian N., Yu S., Chen S., Yang Y., Li M., Gu H., Chen X. (2024). Gasdermin E promotes translocation of p65 and c-jun into nucleus in keratinocytes for progression of psoriatic skin inflammation. Cell Death Dis..

[8] Kızılyel O., Akdeniz N., Metin M.S., Elmas Ö.F. (2019). Investigation of oxidant and antioxidant levels in patients with psoriasis. Turk. J. Med. Sci..

[9] Lu X., Kuai L., Huang F., Jiang J., Song J., Liu Y., Chen S., Mao L., Peng W., Luo Y., Li Y., Dong H., Li B., Shi J. (2023). Single-atom catalysts-based catalytic ROS clearance for efficient psoriasis treatment and relapse prevention via restoring ESR1. Nat. Commun..

[10] Xu J., Chen H., Qian H., Wang F., Xu Y. (2022). Advances in the modulation of ROS and transdermal administration for anti-psoriatic nanotherapies. J. Nanobiotechnol..

[11] Tsvetkov P., Coy S., Petrova B., Dreishpoon M., Verma A., Abdusamad M., Rossen J., Joesch-Cohen L., Humeidi R., Spangler R.D., Eaton J.K., Frenkel E., Kocak M., Corsello S.M., Lutsenko S., Kanarek N., Santagata S., Golub T.R. (2022). Copper induces cell death by targeting lipoylated TCA cycle proteins. Science.

[12] Gao W., Wang X., Zhou Y., Wang X., Yu Y. (2022). Autophagy, ferroptosis, pyroptosis, and necroptosis in tumor immunotherapy. Signal Transduct. Targeted Ther..

[13] Wang Y., Zhang L., Zhou F. (2022). Cuproptosis: a new form of programmed cell death. Cell. Mol. Immunol..

[14] Nafe R., Hattingen E. (2024). Forms of non-apoptotic cell death and their role in Gliomas—presentation of the current state of knowledge. Biomedicines.

[15] Yang S., Li Y., Zhou L., Wang X., Liu L., Wu M. (2024). Copper homeostasis and cuproptosis in atherosclerosis: metabolism, mechanisms and potential therapeutic strategies. Cell Death Discov..

[16] Hu J., Jiang X. (2025). When essential metal elements become culprits-Cuproptosis in focus. Cancer Cell.

[17] Ala S., Shokrzadeh M., Golpour M., Salehifar E., Alami M., Ahmadi A. (2013). Zinc and copper levels in Iranian patients with psoriasis: a case control study. Biol. Trace Elem. Res..

[18] Wacewicz-Muczyńska M., Socha K., Soroczyńska J., Niczyporuk M., Borawska M.H. (2021). Cadmium, lead and Mercury in the blood of psoriatic and vitiligo patients and their possible associations with dietary habits. Sci. Total Environ..

[19] Lin Q., Zhu J., Chen J., Jia S., Nie S. (2023). Significance of cuproptosis- related genes in the diagnosis and classification of psoriasis. Front. Mol. Biosci..

[20] Yu X., Wang Y., Zhang J., Liu J., Wang A., Ding L. (2024). Recent development of copper-based nanozymes for biomedical applications. Adv. Healthcare Mater..

[21] Zhuang J., Midgley A.C., Wei Y., Liu Q., Kong D., Huang X. (2024). Machine-learning-assisted nanozyme design: lessons from materials and engineered enzymes. Adv. Mater..

[22] Ren X., Chen D., Wang Y., Li H., Zhang Y., Chen H., Li X., Huo M. (2022). Nanozymes-recent development and biomedical applications. J. Nanobiotechnol..

[23] Chen W., Li D. (2020). Reactive oxygen species (ROS)-responsive nanomedicine for solving ischemia-reperfusion injury. Front. Chem..

[24] Proksch E. (2018). pH in nature, humans and skin. J. Dermatol..

[25] Ahn R., Gupta R., Lai K., Chopra N., Arron S.T., Liao W. (2016). Network analysis of psoriasis reveals biological pathways and roles for coding and long non-coding RNAs. BMC Genom..

[26] Li J., Wu F., Li C., Sun S., Feng C., Wu H., Chen X., Wang W., Zhang Y., Liu M., Liu X., Cai Y., Jia Y., Qiao H., Zhang Y., Zhang S. (2022). The cuproptosis-related signature predicts prognosis and indicates immune microenvironment in breast cancer. Front. Genet..

[27] Zhang G., Sun J., Zhang X. (2022). A novel cuproptosis-related LncRNA signature to predict prognosis in hepatocellular carcinoma. Sci. Rep..

[28] Kresse G., Hafner J. (1993). Ab initio molecular dynamics for liquid metals. Phys. Rev. B Condens. Matter.

[29] Kresse G., Hafner J. (1994). Ab initio molecular-dynamics simulation of the liquid-metal-amorphous-semiconductor transition in germanium. Phys. Rev. B Condens. Matter.

[30] Perdew J.P., Burke K., Ernzerhof M. (1996). Generalized gradient approximation made simple. Phys. Rev. Lett..

[31] Kresse G., Joubert D. (1999). From ultrasoft pseudopotentials to the projector augmented-wave method. Phys. Rev. B.

[32] Blöchl P.E. (1994). Projector augmented-wave method. Phys. Rev. B Condens. Matter.

[33] Grimme S., Antony J., Ehrlich S., Krieg H. (2010). A consistent and accurate ab initio parametrization of density functional dispersion correction (DFT-D) for the 94 elements H-Pu. J. Chem. Phys..

[34] You M., Jiang Q., Huang H., Ma F., Zhou X. (2023). 4-Octyl itaconate inhibits inflammation to attenuate psoriasis as an agonist of oxeiptosis. Int. Immunopharmacol..

[35] Liu X.L., Dong X., Yang S.C., Lai X., Liu H.J., Gao Y., Feng H.Y., Zhu M.H., Yuan Y., Lu Q., Lovell J.F., Chen H.Z., Fang C. (2021). Biomimetic liposomal nanoplatinum for targeted cancer chemophototherapy. Adv. Sci. (Weinh.).

[36] Gao J., Chen F., Fang H., Mi J., Qi Q., Yang M. (2020). Daphnetin inhibits proliferation and inflammatory response in human HaCaT keratinocytes and ameliorates imiquimod-induced psoriasis-like skin lesion in mice. Biol. Res..

[37] van der Fits L., Mourits S., Voerman J.S.A., Kant M., Boon L., Laman J.D., Cornelissen F., Mus A.-M., Florencia E., Prens E.P., Lubberts E. (2009). Imiquimod-induced psoriasis-like skin inflammation in mice is mediated via the IL-23/IL-17 axis. J. Immunol..

[38] Niu M.-T., Li Q.-R., Huang Q.-X., Qin Y.-T., Meng D., Liang J.-L., Zhang X.-Z. (2025). Amplifying synergistic effects of cuproptosis and bacterial membrane vesicles-mediated photothermal therapy by multifunctional nano-biohybrid for anti-tumor immunotherapy. Adv. Funct. Mater..

[39] Hou C.-C., Chen Q.-Q., Wang C.-J., Liang F., Lin Z., Fu W.-F., Chen Y. (2016). Self-supported cedarlike semimetallic Cu3P nanoarrays as a 3D high-performance janus electrode for both oxygen and hydrogen evolution under basic conditions. ACS Appl. Mater. Interfaces.

[40] Liu J., Yuan Y., Cheng Y., Fu D., Chen Z., Wang Y., Zhang L., Yao C., Shi L., Li M., Zhou C., Zou M., Wang G., Wang L., Wang Z. (2022). Copper-based metal–organic framework overcomes cancer chemoresistance through systemically disrupting dynamically balanced cellular redox homeostasis. J. Am. Chem. Soc..

[41] Chao D., Dong Q., Yu Z., Qi D., Li M., Xu L., Liu L., Fang Y., Dong S. (2022). Specific nanodrug for diabetic chronic wounds based on antioxidase-mimicking MOF-818 nanozymes. J. Am. Chem. Soc..

[42] Feng S., Qiao W., Xia L., Yu L., Lang Y., Jin J., Liu Y., Chen F., Feng W., Chen Y. (2025). Nanoengineered, ultrasmall and catalytic potassium calcium hexacyanoferrate for neuroprotection and temporal lobe epilepsy treatment. Science Bulletin.

[43] Yang X., Li C., Ge M., Li X., Zhao W., Guo H., Nie H., Liu J. (2024). Mn(II)-Aloe-Emodin nanoscale coordination polymer enhances ferroptosis by synergistically enhancing reactive oxygen species generation via the Nrf2-GPX4 axis. Adv. Healthcare Mater..

[44] Zhong D., Yang X., Yang J., Luo Z., Feng Z., Ma M., Liao Y., Tang Y., Wen Y., Liu J., Hu S. (2025). Oxygen vacancy-engineered bimetallic nanozymes for disrupting electron transport chain and synergistic multi-enzyme activity to reverse oxaliplatin resistance in colorectal cancer. J. Nanobiotechnol..

[45] Wu J., Jiao D., Liu Q., Tian B., Liu T., Wu Q., Nan Y., Chang X., Jiang S., Yang Q., Cui X., Chen F. (2024). Atomic interface engineering-mediated metallene nanozyme boosts efficient photothermal catalytic tumor-specific therapy. Adv. Funct. Mater..

[46] Chen L., Min J., Wang F. (2022). Copper homeostasis and cuproptosis in health and disease. Signal Transduct. Targeted Ther..

[47] Lei S., Liu K., Liu C., An R., Bao Z., Zhang H., Zou M., Chen J., Zhang H., Wei L., Lan Z., Chen L. (2025). Cis-resveratrol blocks crystal-induced NLRP3 inflammasome activation via the TRPV4-Ca^2+^-phagocytosis-ROS axis. Phytomedicine.

[48] Wang J., Huang J., Wang L., Chen C., Yang D., Jin M., Bai C., Song Y. (2017). Urban particulate matter triggers lung inflammation via the ROSMAPK- NF-κB signaling pathway. J. Thorac. Dis..

[49] Yang L., Zhao Z., Tian B., Yang M., Dong Y., Zhou B., Gai S., Xie Y., Lin J. (2024). A singular plasmonic-thermoelectric hollow nanostructure inducing apoptosis and cuproptosis for catalytic cancer therapy. Nat. Commun..

[50] Yu C., Chen D., Zhu D., Feng L., Yang L., Berdimurodov E., Zang P., Zhu Y., Hu Y., Sang J., Yang P. (2025). Dual strategy of Ca2+ influx and collagen denaturation to remodel the extracellular matrix and amplify sonopiezoelectric therapy. Adv. Mater..

[51] Dong Y., Dong S., Yu C., Liu J., Gai S., Xie Y., Zhao Z., Qin X., Feng L., Yang P., Zhao Y. (2023). Mitochondria-targeting Cu3VS4 nanostructure with high copper ionic mobility for photothermoelectric therapy. Sci. Adv..

[52] Yu C., Dong Y., Zhu X., Feng L., Zang P., Liu B., Dong S., Zhao R., Xu R., Yang P. (2024). Oxygen vacancy piezoelectric nanosheets constructed by a photoetching strategy for ultrasound “Unlocked” tumor synergistic therapy. Nano Lett..

[53] Zang P., Yu C., Zhang R., Yang D., Gai S., Liu B., Shen R., Yang P., Lin J. (2024). Phase engineered CuxS–Ag2S with photothermoelectric activity for enhanced multienzyme activity and dynamic therapy. Adv. Mater..

[54] Zhang S., Du Y., Yang L., Dong Y., Zang P., Yang M., Gai S., Yang P. (2025). Self-doped Cu2+xZn1-xSnSe4 nanosheets for enhanced thermoelectric catalytic-ferroptotic therapy. Adv. Mater..

