# Supplementary material for: Copper-based nanozymes synergistically enhance Cuproptosis for psoriasis treatment
Source: Mater Today Bio. 2026 Jan 29;37:102855. doi: 10.1016/j.mtbio.2026.102855 (PMC12906090; doi:10.1016/j.mtbio.2026.102855)
Supplement: Multimedia component 1 [file mmc1.docx]

**Supplementary figures**

Figure S1. The hydrated particle size distribution of Cu-NZs.

Figure S2. Elemental mapping of Cu, C and O in Cu-NZs.

Figure S3. Hydrated diameter of Cu-NZs in different solvents. Mean ± SD, n=3.

Figure S3. Thermogravity curves of Cu-NZs.

Figure S4. FTIR patterns of Cu-NZs.

Figure S5. XPS spectra of wide-scan, O 1s.

Figure S6. XPS spectra of wide-scan, C 1s.

Figure S7. Net oxygen generation from H_2_O_2_ catalyzed by different concentrations of Cu-NZs.

Figure S8. Digital photographs of H_2_O_2_ solutions in Figure 1j (2 h after the addition of 100 µg/mL Cu-NZs.)

Figure S9. SOD-like activities of Cu-NZs of varied concentrations. Data were presented as mean ± SD.

Figure S10. UV-vis absorbance spectra of TMB in presence of Cu-NZs (40 μg/mL) with the addition of different concentrations of TMB (50 μM, 100 μM, 200 μM, 400 μM and 800 μM ) and the corresponding digital photos of each group.

Figure S11. POD-mimicking activity of Cu-NZs. 1) Cu-NZs + H_2_O_2_ + TMB, 2) Cu-NZs + TMB, 3) H_2_O_2_ + TMB, and 4) H_2_O_2_)

Figure S12. UV-vis absorbance spectra of TMB in presence of Cu-NZs (40 μg/mL), and H_2_O_2_ (100 mM) with the addition of different concentrations of TMB (50 μM, 100 μM, 200 μM, 400 μM and 800 μM ) and the corresponding digital photos of each group.

Figure S13. UV-vis absorbance spectra of TMB in presence of Cu-NZs (40 μg/mL), and H_2_O_2_ (0 mM, 5 mM, 25 mM, 50 mM, and 100 mM) with the addition of TMB (800 μM ) and the corresponding digital photos of each group.

Figure S14. UV-vis absorbance spectra of TMB in presence of Cu-NZs (0 μg/mL, 5 μg/mL, 10 μg/mL, 20 μg/mL, and 40 μg/mL), and H_2_O_2_ (100 mM) with the addition of TMB (800 μM) and the corresponding digital photos of each group.

Figure S15. SEM images showing the microstructures of (a) gel and (b) Cu-NZs/gel, and (c) Element mapping images of Cu-NZs/gel.

Figure S16. The mRNA expression levels of hif1α treated with Cu-NZs (7.5 and 15 μg/mL) for 24 h. Data are presented as means ± SD from at least three independent experiments. **p < 0.01 and ***p < 0.001

Figure S17. Flow cytometry analysis and quantification of DCFH-DA levels in HaCaT cells treated with Cu-NZs (7.5 and 15 μg/mL) for 24 h. Data are presented as mean ± SD from at least three independent experiments. ****p < 0.0001.

Figure S18. Flow cytometry analysis and quantification of JC-1 monomer formation in HaCaT cells treated with Cu-NZs (7.5 and 15 μg/mL) for 24 h. Data are presented as mean ± SD from at least three independent experiments. ****p < 0.0001.

Figure S19. Cu-NZs inhibited M5 cocktail-induced inflammatory cytokine production in HaCat cells. HaCaT cells were treated with either DMEM (control) or M5 cocktail in DMEM for 24 hours. The mRNA expression levels of inflammatory cytokines—S100A7, S100A8, S100A9, IL-1β, CCL1, and CXCL3—were quantified using qRT-PCR in M5-stimulated HaCaT cells. Data are presented as means ± SD from at least three independent experiments. *p < 0.05, ***p < 0.001 and ****p < 0.0001.

Figure S20. Representative H&E-stained sections of major organs (liver, heart, spleen, lungs, kidneys) after topical Cu-NZ gel treatment.

**Supplementary tables**

Table S1. Results of the fits of the Cu-NZs region, values given in atomic % of total intensity.

Table S2. Results of the fits of the XPS Cu2p region.

Table S3. Results of the fits of the XPS C1s region.

| **Primer** | **Forward (5'-3')** | **Reverse (5'-3')** |
| --- | --- | --- |
| ***Human*** |  |  |
| GAPDH | GTCTCCTCTGACTTCAACAGCG | ACCACCCTGTTGCTGTAGCCAA |
| IL-1β | CACGATGCACCTGTACGA | AACACCACTTGTTGCTCCA |
| S100A7 | CCCAACTTCCTTAGTGCCTGTGAC | GCTCTGCTTGTGGTAGTCTGTGG |
| S100A8 | GAAGGTTCTGTTTTTCAGGTGGG | GAGGACACTCGGTCTCTAGCAA |
| S100A9 | GAACACATCATGGAGGACCTGGAC | GGTTAGCCTCGCCATCAGCATG |
| CCL1 | TGCTGCTCCTGCTCCTGGTAG | GGGGACTTCACGTTCACACTTTGG |
| CXCL3 | TTCACCTCAAGAACATCCAAAGTG | TTCTTCCCATTCTTGAGTGTGGC |
| hif1α | TATGAGCCAGAAGAACTTTTAGGC | CACCTCTTTTGGCAAGCATCCTG |
| DLAT | CAGCTACTCCTGCTGGACCAAA | GGTGATTCTACCATCTGGTCCTG |
| LIAS | GCCAAGAAGGTTCAGCCTGATG | GTCTACATCTGCCTCACGAAGTG |
| FDX1 | CTGGCTTGTTCAACCTGTCACC | GATTTGGCAGCCCAACCGTGAT |
| ***Mouse*** |  |  |
| β-actin | CATTGCTGACAGGATGCAGAAGG | TGCTGGAAGGTGGACAGTGAGG |
| IL-1β | CTTCAGGCAGGCAGTATC | CAGCAGGTTATCATCATCATC |
| IL-6 | CCGCTATGAAGTTCCTCTC | GGTATCCTCTGTGAAGTCTC |
| CXCL3 | GCCACTCTCAAGGATGGTCAAGAAG | GGACTTGCCGCTCTTCAGTATCTTC |

Table S4. The information of primer.
